# Neonatal Gerbode Defect Resulting in Cardiogenic Shock

**DOI:** 10.1016/j.atssr.2023.05.015

**Published:** 2023-06-10

**Authors:** Lillian Kang, Anna C. Hoover, Michael Camitta, Joseph W. Turek, Nicholas D. Andersen

**Affiliations:** 1 Department of Surgery, Duke University Medical Center, Durham, North Carolina; 2 Duke University Medical School, Durham, North Carolina; 3 Duke Children’s Pediatric & Congenital Heart Center, Duke University Medical Center, Durham, North Carolina

## Abstract

Congenital Gerbode defects, consisting of a deficiency in the membranous septum causing left ventricle–to–right atrium shunting, are rarely hemodynamically significant. Here, we present the case of a neonate with a large unrestrictive Gerbode defect, patent foramen ovale, patent ductus arteriosus, and pulmonary valve insufficiency resulting in a circular intracardiac shunt and cardiogenic shock. The patient was managed with venoarterial extracorporeal membrane oxygenation followed by neonatal Gerbode defect repair. After repair, the patient had an uncomplicated postoperative course. To our knowledge, a neonatal congenital Gerbode defect resulting in cardiogenic shock is exceedingly rare.

In 1958, Gerbode first described the repair of an anatomic defect in the membranous septum causing left ventricle–to–right atrium shunting.^1^ These defects make up <1% of congenital cardiac defects and 0.08% of intracardiac shunts.^2^^,^^3^ Whereas most Gerbode defects are congenital, acquired Gerbode defects can result from cardiac surgery, percutaneous cardiac interventions, endocarditis, and myocardial infarction.^4^ Gerbode defects are classified by the location of the septum defect relative to the tricuspid valve septal leaflet^5^: supravalvular, infravalvular, or intermediate with both supravalvular and infravalvular components. Approximately 81% of Gerbode defects are supravalvular.^6^ Symptomatic Gerbode defects are usually manifested with dyspnea.^6^ Here we describe the unique case of a Gerbode defect discovered in a neonate resulting in cardiogenic shock.

The patient is a full-term male neonate who was born through uncomplicated spontaneous vaginal delivery with Apgar scores of 9 and 9 and birthweight of 3.24 kg. Because of prenatally diagnosed ventricular septal defect and pulmonary stenosis, he was prescribed a prostaglandin infusion (0.01 μg/kg per minute). No other postnatal complications or interventions were required, and the patient was transferred in stable condition to the pediatric cardiac intensive care unit on room air. Postnatal transthoracic echocardiography (TTE) revealed a unicuspid and severely dysplastic pulmonary valve, right ventricular hypertrophy, large supravalvular Gerbode defect with no ventricular septal defect or tricuspid regurgitation, and patent foramen ovale (PFO) with bidirectional shunting (Figure 1). The patient’s newborn screen and karyotype were normal, and he tested negative for Noonan syndrome. On day 2 of life, given pulmonary stenosis (pressure gradient of 50 mm Hg) and ductal-dependent pulmonary blood flow, the patient underwent cardiac catheterization, which revealed a thickly hypertrophied right ventricle with normal systolic function, small functional right ventricular cavity due to hypertrophy, suprasystemic right ventricular pressures of 82/8 mm Hg, and severely thickened and dysplastic-appearing pulmonary valve with some doming of the leaflets and a 7-mm pulmonary valve annulus anterior-posterior diameter. Percutaneous pulmonary valve balloon valvuloplasty was performed with an 8-mm × 2-cm Tyshak II balloon (BVM Medical) with subsequent pressure gradient of 23 mm Hg and moderate to severe pulmonary regurgitation. Postintervention right atrial mean pressure was 6 mm Hg and right ventricular end-diastolic pressure was 12 mm Hg. Postintervention systemic saturation was 89% and mixed venous saturation was 74%, suggesting a mild right-to-left shunt at atrial level as no ventricular septal defect was detected. After pulmonary valvuloplasty, prostaglandin infusion was discontinued.

**Figure 1:**
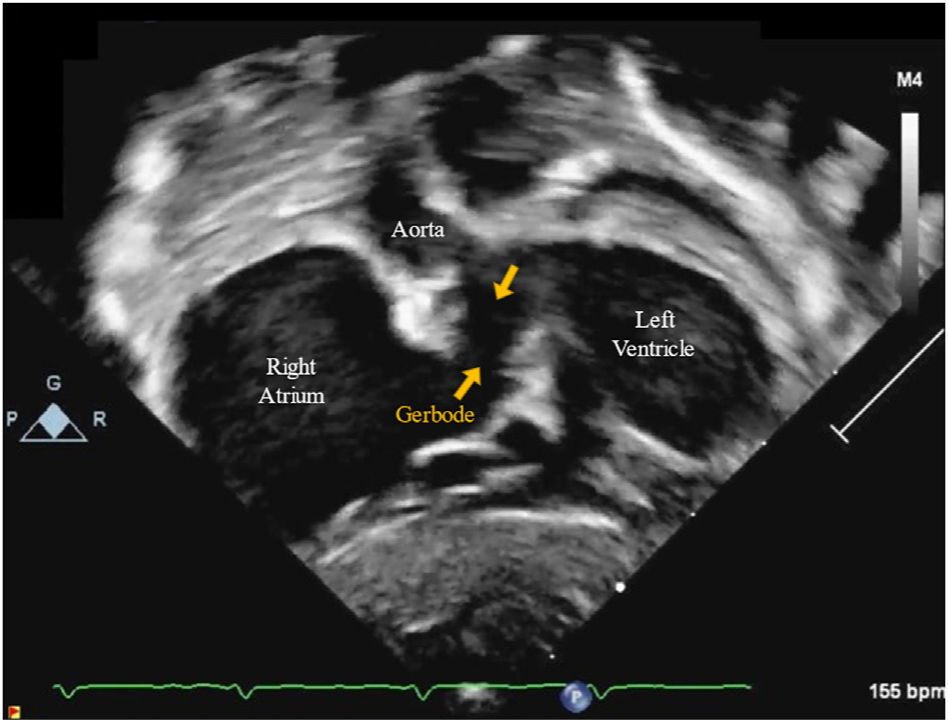
Transthoracic echocardiography demonstrating patient’s Gerbode defect (arrows).

The following day, the patient had evidence of cardiogenic shock with refractory hypotension and lactic acidosis (peak lactate level of 10.9 mmol/L) despite increasing inotropic support (epinephrine, 0.15 μg/kg per minute; norepinephrine, 0.1 μg/kg per minute; vasopressin, 40 mU/kg per hour). It is likely that pulmonary valve balloon valvuloplasty resulted in the patient’s hemodynamic instability because of relief of severe pulmonary stenosis and subsequent iatrogenic pulmonary regurgitation, improvement in right ventricular pressure, and worsening of the intracardiac shunt. The decision was made to proceed with venoarterial extracorporeal membrane oxygenation (ECMO) cannulation and an attempt at decreasing left-to-right shunting and improving effective systemic cardiac output by patent ductus arteriosus (PDA) ligation. Before ECMO cannulation, the PDA was ensnared with a vessel loop and occluded. This did not result in any hemodynamic change, so the patient was centrally cannulated for ECMO with 8F aortic and 18F right atrial cannulas with flows of 100 mL/kg per minute, which allowed the weaning off of inotropes.

Because PDA ligation did not meaningfully improve the patient’s hemodynamic status, the cause of the patient’s cardiogenic shock was suspected to be due primarily to the large unrestrictive Gerbode defect resulting in an intracardiac circular shunt (left ventricle, to right atrium, to left atrium, to the left ventricle), functionally mimicking severe mitral regurgitation and resulting in poor effective systemic cardiac output. After multidisciplinary discussions, it was determined that repair of the Gerbode defect to eliminate the intracardiac circular shunt would serve the patient best.

After the patient was stabilized, he was taken for Gerbode defect repair on day 5 of life. A large Gerbode defect was identified intraoperatively posterior to the tricuspid valve rising above the plane of the tricuspid valve (Figure 2; Video). This defect was closed with a circular patch of bovine pericardium patch in running fashion with 6-0 Prolene (Figure 3). The tricuspid valve was tested and found to be competent. A large PFO was identified and subtotally closed, leaving a 3-mm defect. The total cross-clamp time was 45.8 minutes, and total cardiopulmonary bypass time was 77 minutes. Postrepair transesophageal echocardiography showed no residual Gerbode defect, the small residual PFO, and free pulmonary insufficiency without stenosis. The patient’s immediate postoperative course was uncomplicated. On postoperative day 4, the patient was weaned from inotropic support and extubated to high-flow nasal cannula. The patient was transferred to stepdown on postoperative day 6. He was weaned off supplemental oxygen to room air on postoperative day 12. By the time of discharge, he was tolerating full bolus enteral feeds through nasogastric tube. Discharge occurred on postoperative day 13, and discharge TTE again demonstrated lack of residual Gerbode defect with mild pulmonary valve stenosis (pressure gradient of 29 mm Hg). His most recent TTE 2 months postoperatively demonstrated lack of Gerbode defect and mild pulmonary valve stenosis (pressure gradient of 26 mm Hg; Figure 3). The patient was most recently seen in clinic at 7 months postoperatively, at which time he is gaining appropriate weight through oral intake and remains on room air.

**Figure 2:**
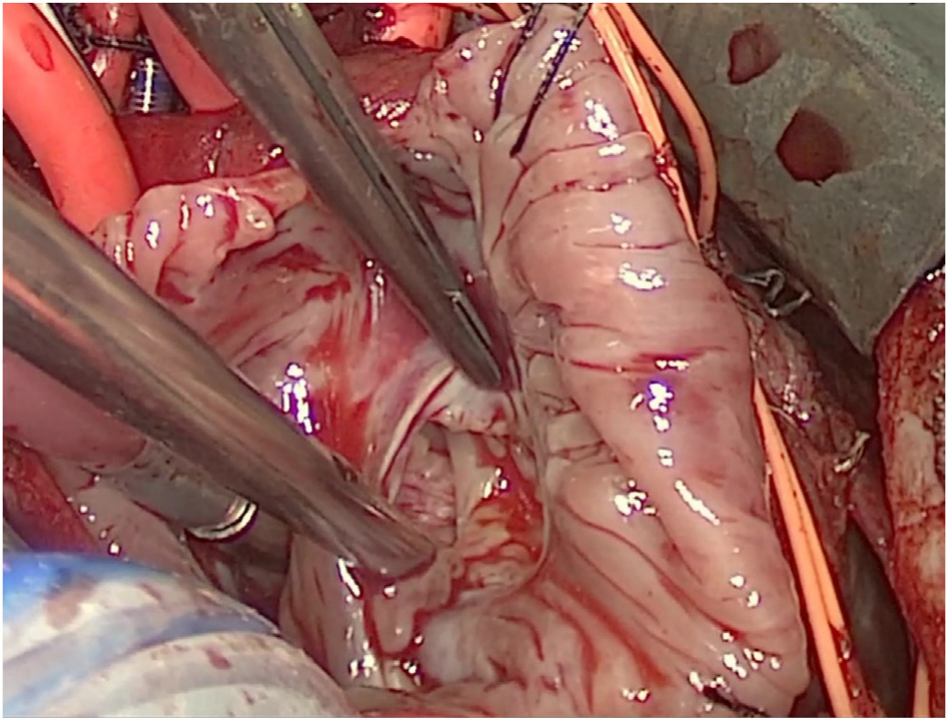
Intraoperative picture of the patient’s Gerbode defect.

**Figure 3:**
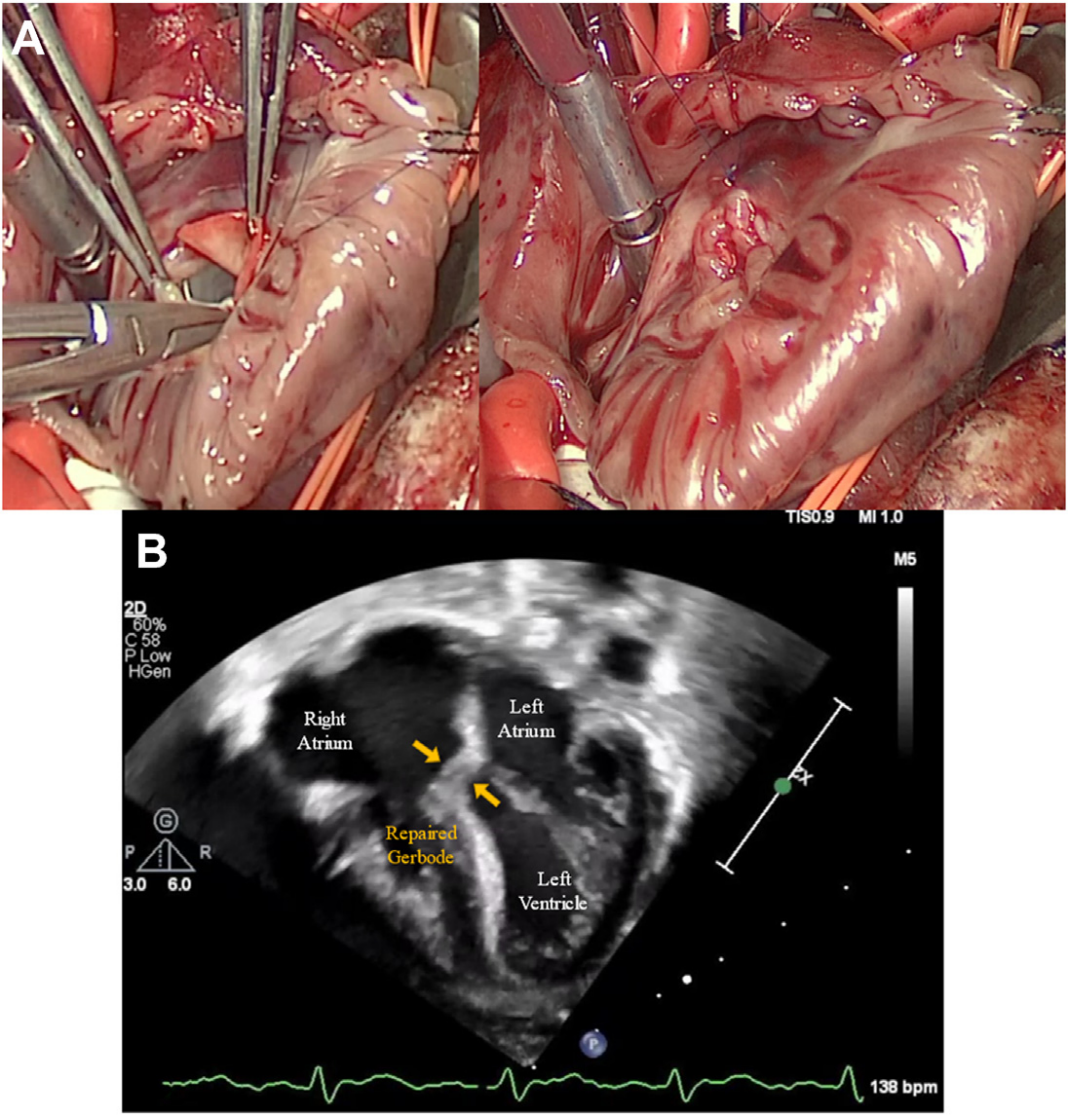
(A) Intraoperative picture of the Gerbode defect repair. (B) Postoperative transthoracic echocardiogram of repaired Gerbode defect (arrows).

## Comment

A neonatal congenital Gerbode defect resulting in cardiogenic shock is exceedingly rare. This patient’s unique constellation of congenital heart defects including large Gerbode defect, PFO, PDA, and pulmonary valve insufficiency after balloon dilation resulted in an unfortunate circular intracardiac shunt reminiscent of severe mitral regurgitation. After Gerbode defect repair, the patient had a relatively uncomplicated postoperative course.
